# Image Processing of Mg-Al-Sn Alloy Microstructures for Determining Phase Ratios and Grain Size and Correction with Manual Measurement

**DOI:** 10.3390/ma14175095

**Published:** 2021-09-06

**Authors:** Ali Ercetin, Fatih Akkoyun, Ercan Şimşir, Danil Yurievich Pimenov, Khaled Giasin, Manjunath Patel Gowdru Chandrashekarappa, Avinash Lakshmikanthan, Szymon Wojciechowski

**Affiliations:** 1 Department of Mechanical Engineering, Faculty of Engineering and Architecture, Bingol University, Bingöl 12000, Turkey; 2 Department of Mechanical Engineering, Faculty of Engineering, Aydın Adnan Menderes University, Aydın 09010, Turkey; fatih.akkoyun@adu.edu.tr; 3 Department of Transportation Services, Sultandağı Vocational School, Afyon Kocatepe University, Afyonkarahisar 03900, Turkey; esimsir@aku.edu.tr; 4 Department of Automated Mechanical Engineering, South Ural State University, Lenin Prosp. 76, 454080 Chelyabinsk, Russia; danil_u@rambler.ru; 5 School of Mechanical and Design Engineering, University of Portsmouth, Portsmouth PO1 3DJ, UK; Khaled.giasin@port.ac.uk; 6 Department of Mechanical Engineering, PES Institute of Technology and Management, Visvesvaraya Technological University, Belagavi 590018, India; manju09mpm05@gmail.com; 7 Department of Mechanical Engineering, Nitte Meenakshi Institute of Technology, Bengaluru 560064, India; avinash.laks01@gmail.com; 8 Faculty of Mechanical Engineering, Poznan University of Technology, 60-965 Poznan, Poland; sjwojciechowski@o2.pl

**Keywords:** microstructure, grain size, computer vision, automated counting, intermetallic phases, image processing

## Abstract

The study of microstructures for the accurate control of material properties is of industrial relevance. Identification and characterization of microstructural properties by manual measurement are often slow, labour intensive, and have a lack of repeatability. In the present work, the intermetallic phase ratio and grain size in the microstructure of known Mg-Sn-Al alloys were measured by computer vision (CV) technology. New Mg (Magnesium) alloys with different alloying element contents were selected as the work materials. Mg alloys (Mg-Al-Sn) were produced using the hot-pressing powder metallurgy technique. The alloys were sintered at 620 °C under 50 MPa pressure in an argon gas atmosphere. Scanning electron microscopy (SEM) images were taken for all the fabricated alloys (three alloys: Mg-7Al-5Sn, Mg-8Al-5Sn, Mg-9Al-5Sn). From the SEM images, the grain size was counted manually and automatically with the application of CV technology. The obtained results were evaluated by correcting automated grain counting procedures with manual measurements. The accuracy of the automated counting technique for determining the grain count exceeded 92% compared to the manual counting procedure. In addition, ASTM (American Society for Testing and Materials) grain sizes were accurately calculated (approximately 99% accuracy) according to the determined grain counts in the SEM images. Hence, a successful approach was proposed by calculating the ASTM grain sizes of each alloy with respect to manual and automated counting methods. The intermetallic phases (Mg_17_Al_12_ and Mg_2_Sn) were also detected by theoretical calculations and automated measurements. The accuracy of automated measurements for Mg_17_Al_12_ and Mg_2_Sn intermetallic phases were over 95% and 97%, respectively. The proposed automatic image processing technique can be used as a tool to track and analyse the grain and intermetallic phases of the microstructure of other alloys such as AZ31 and AZ91 magnesium alloys, aluminium, titanium, and Co alloys.

## 1. Introduction

Magnesium (Mg) is the eighth-most abundant lightweight structural material found in the earth’s crust and is the third most plentiful element dissolved in seawater [1,2]. Mg material possesses a low density of 1.74 g/cm3, which is ≈22% steel and ≈65% aluminium, and is most comparable to fibre-based composites and plastics [3,4]. Thermal stability, damping characteristics, mechanical properties, low density coupled with good electromagnetic shielding, and machinability are a few of the characteristic features of magnesium that allow it to replace other metals on a large scale [5,6,7,8]. Owing to their excellent properties, industries are fabricating parts that are useful for automotive, aircraft, and military devices; biomedical implants; smartphones; computers; and household appliances, etc. [9]. Environmental perspectives also demand the use of magnesium parts that reduce vehicle weight, with design changes in structures and engine size that could result in fuel savings and reduce greenhouse gas emissions (80% of CO_2_ emissions from road transport and 45% from cars) by 60% [10,11]. Significant attention should be given to enhance certain properties that scale up the production and, in turn, the applications of magnesium parts.

In recent years, worldwide attention with intense research and development activities has led to the introduction of novel materials that exhibit superior properties with the addition of alloying elements and synthesis methods [12,13]. Table 1 shows the addition of alloying/reinforcing elements to magnesium and its alloys that have resulted in better mechanical, thermal, tribological, and microstructure properties. Mg alloys are synthesized by various processing routes, such as DMD (Direct Metal Deposition), SLM (Selective Laser Melting), casting, and powder metallurgy [12,14]. In the cast processing route, the defects (porosity, shrinkage, hot tears, and segregations), material wastages, and the fact that molten magnesium is more prone to oxidation and burn limit the extensive use of casting processes [15,16]. Secondary processing methods (such as plastic deformation and heat treatment methods) are applied to the cast products to limit the casting defects and enhance the product’s properties [17,18,19,20]. The secondary processes result in a high manufacturing cost of alloy development and the fabrication of parts [21]. Powder metallurgy proved to be the most promising technique to yield fine grain structures with superior properties in the significant alloys compared to the casting processing route [9,22]. In addition, its greater flexibility in fabricating complex parts with the desired geometrical accuracy ensures near-net-shaped PM parts [23]. The hot pressing in powder metallurgy technique reduces both sintering temperature and time; refines the grain structure and ensures grain uniformity; improves density and strength; is free from microstructural cracks; and has greater simplicity, flexibility, and low cost and can therefore offer parts of a better dimensional stability [24,25,26].

Alloying elements (Gd, Al, Sn) are known to improve the ductility and grain structure in Mg alloys [28,31]. Refined grain structure with alloying elements (Sn, Al, and Si) ensures higher wear resistance in Mg components [38,39,40]. Al, Zn, Sn, and Ca are the family of light metal groups used as the major alloying elements for Mg alloys [35,41,42]. The addition of Ca, Mn, and Sn elements to Mg alloys (Mg-9Al and AZ31) refines grain structures and thereby enhances corrosion resistance [30]. The addition of Al and Sn improves the ductility in powder metallurgy parts [31,36]. The literature review confirmed that the appropriate control of microstructure with alloy selection and processing methods resulted in better properties, i.e., hardness [7], strength [27,43], wear resistance [38], and corrosion resistance [30], in Mg and its alloys. In addition, the formability [28] and machinability [44,45] of magnesium components are also influenced by the microstructure. The above literature confirms that PM based on hot pressing is a promising technique which offers components with complex geometry for a wide range of alloys with refined microstructure [9]. Furthermore, appropriate control of microstructures with alloys resulted in improved mechanical, thermal, and tribological properties. Therefore, a suitable method needs to be established that estimates the changes in microstructure with differences in chemical composition or production methods.

To date, X-ray analysis, optical, and scanning electron microscopy are the three most popular techniques applied by industrial and research practitioners to analyse microstructures [46,47,48]. In Mg alloys, the grain size in microstructures greatly influences mechanical and other tribological properties [27,33]. Examining the grain sizes in microstructures is of primary importance. Compared to coarse grain structures, the number of grains per unit area in a fine-grained microstructures is greater [49]. According to the Hall–Petch effect, the increased length of grain boundaries creates difficulty in dislocation movements, thereby improving the mechanical properties of the material [49,50,51]. Scanning electron micrographs are taken and analysed manually to assess the total number of grains and length of grain boundaries present in microstructures [9,52]. The manual method in estimating the microstructural features (e.g., grain size, length of grain boundaries, number of grains) is time-consuming, laborious, requires expert materials scientists, and suffers from poor repeatability [53,54]. Therefore, the development of an accurate method that could quickly detect the microstructure features via image analysis techniques is of industrial relevance [55,56,57].

Computer vision technology reinforced with machine learning has been enhanced for various practices and has demonstrated noteworthy findings [58]. Object detection (2D and 3D) and classification processes are one of these major fields in which different features, such as object edges, primitive shapes, and colour scale, are differentiated using image and video streams [59,60,61]. To accomplish certain operations, machine learning algorithms have been combined with object classification models. These include: logistic regression (LR), support vector machine (SVM), random forest (RF), decision tree (DT), k-nearest neighbours (kNN), multilayer perceptron (MLP), and Naïve Bayes (NB), which have been proven effective in a variety of applications, ranging from micro-scale to macro-scale characterization [62,63]. Most of the studies published in the field of vision-based object investigation agree that characterization, image resolution, image processing methods, and colour scale represent the critical parameters of the process [64,65]. Today, with the help of advanced computer vision technology, image processing techniques have been developed to capture the morphological features (grain size, volume fractions of grains, colony size, etc.) for analysis of the complex microstructure of Ti6Al4V alloys [54]. The microstructure features of high carbon steels have been quantified to identify the carbide network and pearlite matrix using digital image processing techniques [66]. The optimal image technique successfully detects the presence or lack of fusion defects (created artificially) in powder bed fusion parts [67]. Machine learning techniques can successfully detect and classify the six surface defects in hot-rolled steel parts based on images [68]. The computer vision (CV) technique has been applied to capture the signs of microstructural features and classify them automatically into groups with high accuracy using relatively small data sets [69,70,71]. CV techniques have been used to capture and classify the microstructural features of ultra-high carbon steels treated at different heat treatment conditions [72]. CV (autonomous, objective, and repeatable) and machine learning algorithms have been successfully applied for the feature detection of microstructures, which ensures clustering, comparison, and analysis of the powder micrographs [73,74]. The performance of materials is analysed based on microstructure as they can detect defects, phases, materials, and so on, with the help of the CV technique [75]. CV technology has been successfully applied to examine the pore morphology in additive manufactured parts [76]. Therefore, CV has proven its potential in evaluating microstructures (to capture information regarding defects, count grains, grain boundaries, properties, and so on from the images retrieved from SEM and OM) by applying filters, thresholds, and mathematical functions.

In the present work, the microstructures of different proportions of alloying elements (Sn and Al) to pure Mg were examined. Novel Mg-Al-Sn alloys were developed using powder metallurgy processing techniques, followed by hot pressing. Three samples prepared at different mixing ratios were examined based on their microstructures. An image processing technique was applied to determine the American Society for Testing and Materials (ASTM) grain size of a number of alloys. CV technology ensures automatic detection of the microstructural (grain size and ASTM grain size number) features of Mg-alloys. The C++ programming language and open-sourced image processing library (OpenCV) is useful for analysis purposes. All the collected micrographs were subjected to the same illumination condition using a microscopic camera, which ensures accurate analysis. The images were processed with colour transformation, thresholding, and contour detections. The processed images were sufficient to accurately predict the grain size and ASTM grain size numbers. The results of the proposed CV-based method were corrected with the manual method. The ASTM grain size numbers were obtained at approximately 99% accuracy. The intermetallic phases (Mg_17_Al_12_ and Mg_2_Sn) were also detected by theoretical calculations and automated measurements. The present study introduces the application of automated image processing procedures for analysing the grain and intermetallic phases of the microstructure with a single image input. The automated procedures offer multiple image outputs, including grain and phase images of Mg-Sn-Al alloys, along with their properties, such as area, perimeter, and counts. Thus, the processed image outputs offer the possibility to characterize SEM images concerning the grains and phase properties of Mg-Sn-Al alloys with a quick, easy, and accurate method. In addition, the present study would be a convenient solution for examining the microstructure of other known metals, such as Ti, Cu alloys, and steels, by computer vision technology.

## 2. Materials and Methods

### 2.1. Material Production

In the present study, micro-sized Mg, Al, and Sn powders (AlfaAesar, Kandel, Germany) with high purities were used. The particle size and purity properties of the powders are given in Table 2. Table 2 presents the details of powder mix composition, sintering temperature, and applied pressure for preparing Mg alloys. The mixing ratios of the powders, measured in terms of weights, are presented in Table 2. The samples were prepared using the production methods followed in the published literature [7,34,35]. The appropriate weight proportions of different powders were mixed and transferred to graphite moulds, followed by sintering at 620 °C and hot-pressing at 50 MPa. It should be noted that argon gas was passed through the hot-pressing system during sintering with a flow rate of 6 l/min to prevent oxidation. Mg alloys with a different weight proportion of Al, Sn, and Mg were produced to determine the accuracy of the CV.

### 2.2. Manual Measurements and Theoretical Calculations

**Figure 1:**
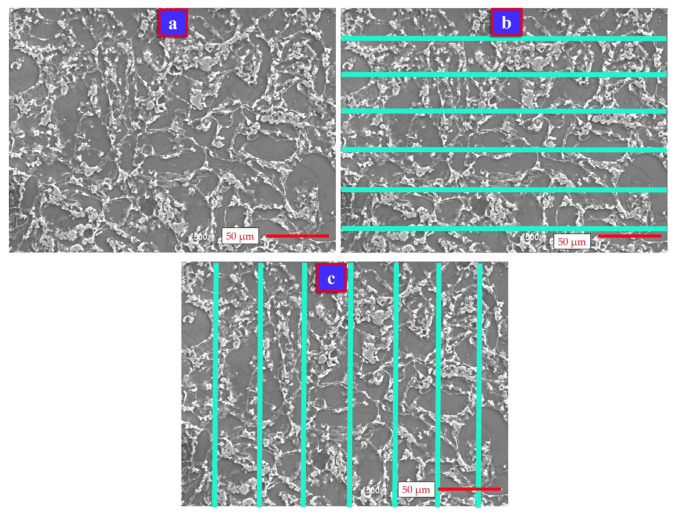
Manual grain counting method: (**a**) original SEM image of sample 1, (**b**) measuring by horizontal, (**c**) measuring by vertical.

**Figure 2:**
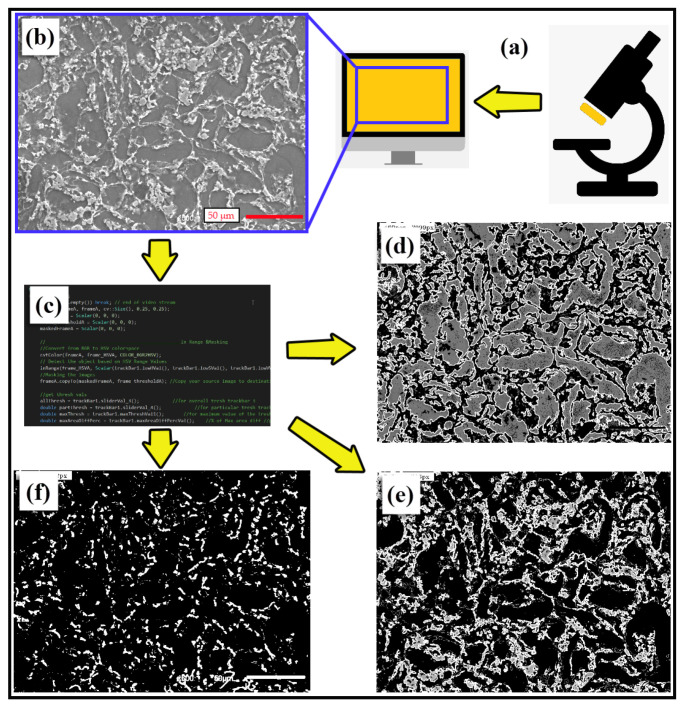
(**a**) Taking SEM image, (**b**) SEM image and image processing procedure outputs; (**c**) software codes, (**d**) grains, (**e**) Mg_17_Al_12_ phases, (**f**) Mg_2_Sn phases.

The prepared Mg-alloys were subjected to sanding, polishing, and etching processes. SEM images (Jeol brand, JSM 6510 model, Jeol Ltd., Tokyo, Japan) at 500× magnification were taken from each Mg-alloy, whose grain boundaries became clear as a result of the etching process. Grain sizes in the microstructures were measured manually using SEM images (see Figure 1) and by the CV method (see Figure 2).

Manual measurements were made on the SEM image with 500× magnification (Figure 1a). Snagit (2018 version, TechSmith Corporation, Okemos, MI, USA) and screen calliper (version 3.3, Iconico Company, Philadelphia, PA, USA) softwares were used for manual counting. Firstly, 6 blue coloured lines with equal lengths were drawn horizontally on the SEM image (Figure 1b). The length of the horizontal lines (LH (µm)) was determined with reference to the actual length of the scale bar. The number of grains along the horizontal (HNG), where any horizontal line crossed the grains, was counted manually. The grains on the edge were counted as half grains. The average grain size along the horizontal (HGS) of the grains, counted with reference to the horizontal lines, was calculated according to Equation (1). Seven blue coloured lines with equal lengths were drawn vertically on the SEM image, as shown in Figure 1c. The length of the vertical lines (LV (µm)) was also determined with reference to the actual length of the scale bar. The number of grains in the vertical (VNG), where any vertical line crossed the grains, were counted manually. The average grain size in the vertical (VGS) of the grains, counted with reference to the vertical lines, was calculated according to Equation (2). The average area of a grain (AAG) of the relevant SEM image was calculated according to Equation (3). To determine the real values of the aspect (X–Y) measurements of the SEM images, the scale bar was taken as a reference. The total number of grains (TN) and total area (TA) were calculated using Equations (4) and (5), respectively. X (mm) is the horizontal length of the SEM image, and Y (mm) is the vertical length of the SEM image. By converting the X and Y lengths from mm to inches, the area (A) of the SEM image in inch^2^ units was calculated using Equation (6). Equation (7) was used to calculate the grain counts per square inch area (G) according to 100× magnification using the total number of grains (TN) counted in the SEM image. Using Equation (8), ASTM grain size numbers (M) of the alloys were calculated.

(1) $$\mathrm{HGS}\;(\mathrm{mm})=(\sum_{1}^{6}\frac{\mathrm{LH}}{\mathrm{HNG}})/(6\times 1000)$$

(2) $$\mathrm{VGS}\;(\mathrm{mm})=(\sum_{1}^{7}\frac{\mathrm{LV}}{\mathrm{VNG}})/(7\times 1000)$$

(3) $$\mathrm{AAG}\;({\mathrm{mm}}^{2})=(\mathrm{HGS}\times \mathrm{VGS})$$

(4) $$\mathrm{TA}\;({\mathrm{mm}}^{2})=X\times Y$$

(5) $$\mathrm{TN}=\frac{\mathrm{TA}}{\mathrm{AAG}}$$

(6) $$A\;({\mathrm{inch}}^{2})=(\frac{X}{25.4})\times (\frac{Y}{25.4})$$

(7) $$G={\left(\frac{500}{100}\right)}^{2}\times \left(\frac{TN}{A}\right)$$

(8) $$M=(\frac{\ln\left(G\right)}{\ln\left(2\right)})+1$$

### 2.3. Parameters and Block Diagram for the Computer Vision Method

In the present study, computer vision software was developed using the C++ programming language and the open-sourced computer vision library (OpenCV) together, which offers a fast and user-friendly method for measuring the grain size and amount of intermetallic phases together by requiring only one SEM image as the input to characterize microstructures with high accuracy. OpenCV is a very extensive image processing library and open-sourced option for CV technology applications [77]. The image processing procedures (Figure 2a–c) were developed to characterize microstructures on an SEM image. The number of grains and ASTM grain size number were calculated with the help of characterization steps. The image processing method starts with image acquisition, followed by pre-processing. In the first step, the actual image (Figure 2b) is converted from red green blue (RGB) to the Hue Saturation Value (HSV) and threshold to minimize the saturation and shadowing effects. In the second step, the image is reduced to a grayscale image, and another thresholding process is applied. In the next step, a contouring operation is processed on the image for determining the contours of the grains. The image is then divided into three parts (Figure 2d–f). These parts are produced by the software for determining the grains (Figure 2d) and grain boundaries (Mg_17_Al_12_ and Mg_2_Sn phases (Figure 2e,f, respectively)). The image processing parameters are defined by the user at the beginning of the process, and the same parameters are applied to all SEM images. In the experiments, the grains are counted using automated methods. To calculate ASTM grain size numbers by the automated method, the number of counted grains used, according to Equations (6)–(8) are applied for the CV method. CV technology also uses additional features, such as the area of grains and grain boundaries, to count their numbers (refer to Figure 2).

**Figure 3:**
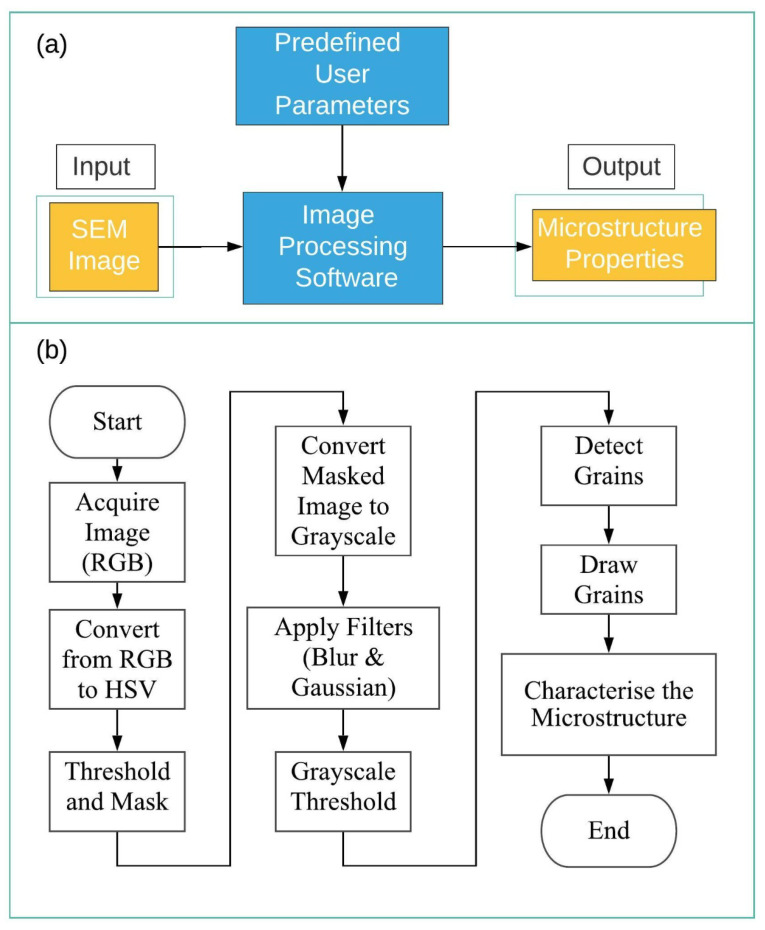
(**a**) Image processing software structure, **(b**) Flowchart of the algorithm.

The software algorithm and flowchart are given in Figure 3a,b, respectively. The flowchart presents the image processing procedure steps. The procedure starts with an image acquisition process. An RGB image is obtained and assigned to a matrix variable. The image is converted to the HSV form, and a threshold is applied for eliminating the noise effects. A normalized box filter and Gaussian filters are used to smooth the images. Image backgrounds are removed with the in-range operation on HSV images. A masking process iss employed to obtain the smoothed actual image background, saturation, and shadows. In the next stage, the image is converted to grayscale and the threshold process is applied before the contour detection operation.

Grain interiors (α-Mg phases) and grain boundaries (Mg_2_Sn and Mg_17_Al_12_ phases) in the SEM images are separated using image processing methods concerning pre-defined user parameters and proposed image processing techniques. The grain interiors and grain boundaries are circulated and numbered with respect to the detected contour parameters. Interiors and boundaries are circulated using the area and perimeter parameters of each detected contour to calculate intermetallic phases in grain boundaries, grain number, and grain size, concerning circulated area and circle count. Thus, image processing outputs were obtained and calculated for grain numbers, areas of grain interiors, and grain boundaries. In addition, there are two different intermetallic phases (Mg_17_Al_12_ and Mg_2_Sn) in the grain boundaries which could be separated from each other automatically by the image processing software.

## 3. Results and Discussions

**Figure 4:**
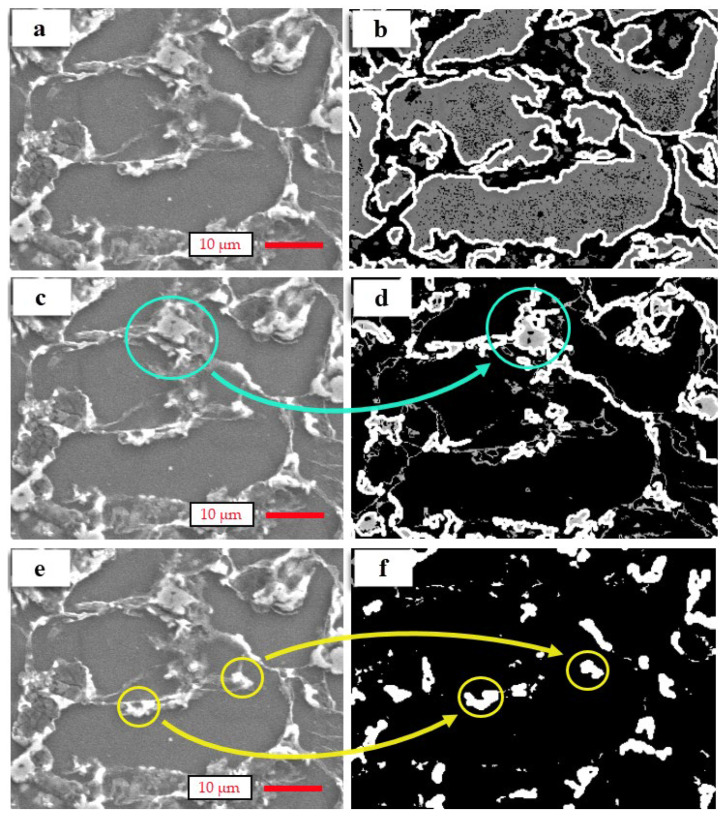
Outputs of image processing of Sample 1: (**a**) SEM image for grain interior, (**b**) processed image for determining grains (α-Mg phase), (**c**) SEM image for Mg_17_Al_12_ phases, (**d**) processed image of Mg_17_Al_12_ phases, (**e**) SEM image for Mg_2_Sn phases, (**f**) processed image of Mg_2_Sn phases.

Figure 4 shows the actual and processed SEM images of Sample 1. In the literature [7,35,63,64,65,66], the microstructure of Mg alloys (containing Mg, Sn, and Al), α-Mg, Mg_2_Sn, and Mg_17_Al_12_ phases were determined by SEM and XRD analysis. The microstructure of Mg alloys (containing Mg, Sn, and Al) includes two intermetallic phases (Mg_2_Sn and Mg_17_Al_12_) in the grain boundaries. According to relevant studies in the literature [7,34,35,63], the grains are dark grey in colour. The Mg_2_Sn phases are white-coloured, and the Mg_17_Al_12_ phases are grey-coloured. In the present study, the same results were observed from actual images (Figure 4a,c,e). In Figure 4, there is only one SEM image as the input, named Sample 1. Sample 1 has three outputs due to the automated measurements. These are the α-Mg phase, the Mg_17_Al_12_ phase, and the Mg_2_Sn phase, respectively. Figure 4 shows how the automated software measured the intermetallic phases (Mg_17_Al_12_ and Mg_2_Sn phases) regarding grain boundaries and determined the grain interiors (α-Mg phase). In the experiments, the SEM images were processed using CV technology based on the proposed method. The determined grains are indicated in Figure 4b; the grains are grey-coloured and the borders of the grains are white-coloured. The grain boundaries are black-coloured. In the processed image for determining Mg_17_Al_12_ phases (Figure 4d), grains and Mg_2_Sn phases are black-coloured and Mg_17_Al_12_ phases are white-coloured (see the areas in Figure 4b,d surrounded with blue circles). Finally, in Figure 4f, grains and Mg_17_Al_12_ phases are black-coloured and Mg_2_Sn phases are white-coloured (see the areas in Figure 4e,f surrounded with yellow circles).

**Figure 5:**
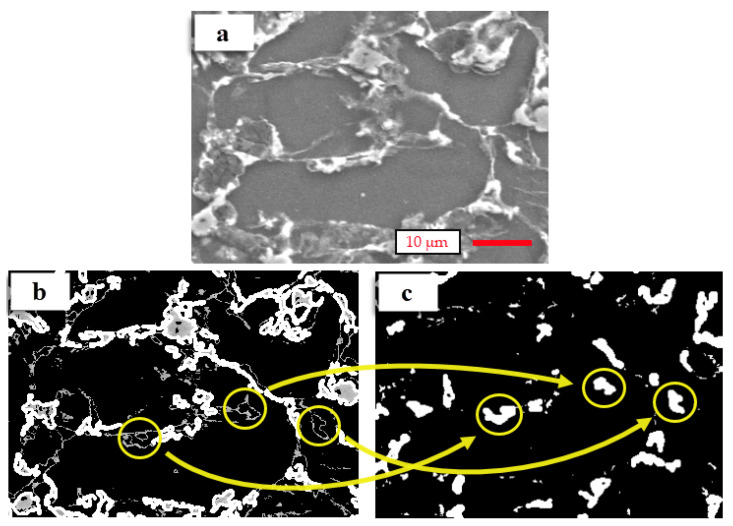
Comparison of the regions of grain boundaries on (**a**) the actual SEM image, (**b**) Mg_17_Al_12_ phases in the processed image, and (**c**) Mg_2_Sn phases in the processed image.

The two intermetallic phases are accurately determined in the grain boundaries. The actual SEM processed images, including the Mg_17_Al_12_ and Mg_2_Sn phases, are demonstrated in Figure 5a–c, respectively. The determined Mg_17_Al_12_ and Mg_2_Sn phases are shown in Figure 5b,c, respectively. According to the obtained results, the CV method accurately detects and separates the two phases from the actual images.

**Figure 6:**
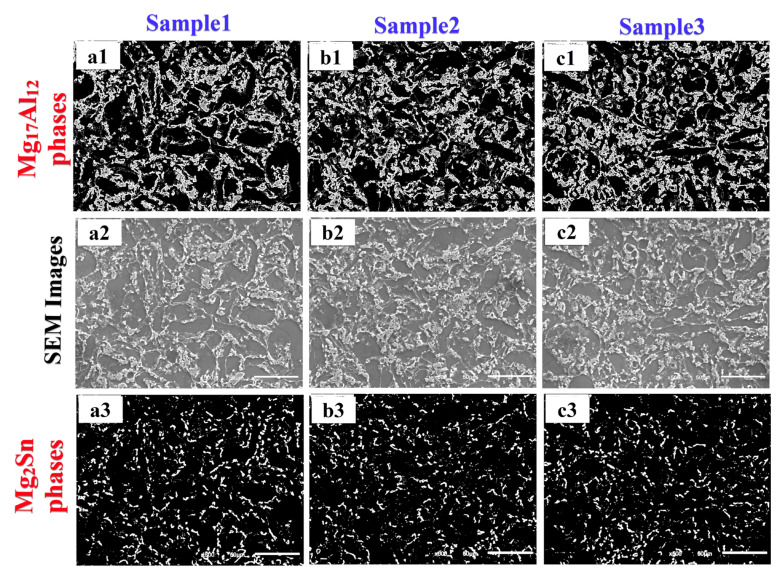
Determined intermetallic phases using CV technology for samples 1, 2, and 3 (horizontal ones refer SEM images and phases; vertical ones refer Samples 1, 2 and 3; 500× magnification, scale bars with 50 µm).

In Figure 6, the SEM and processed images show the homogeneous distribution of the intermetallic phases observed in the microstructure. The actual images in Figure 6a2,b2,c2 were processed using the CV method and were the threshold to separate two intermetallic phases from the actual images. The extracted images from the actual images for the Mg_17_Al_12_ phases are also shown in Figure 6a1,b1,c1, and the Mg_2_Sn phases are similarly shown in Figure 6a3,b3,c3. In Figure 6a1,b1,c1, white colours indicate Mg_17_Al_12_ phases, and the rest are black. It can be observed that the visible area of the Mg_17_Al_12_ phases also increases in the samples by the effect of increasing Al content (Figure 6a1,b1,c1, respectively). For Figure 6a3,b3,c3, white colours indicate Mg_2_Sn phases, and the rest are black-coloured. No visible changes were detected for the Mg_2_Sn phases. According to the experiments, the effect of Al content on the microstructure can be identified from the visible observations. However, it is not easy to determine and express the differences accurately. In contrast, the CV method detects and calculates the effect of Al content in the microstructure and differentiates the two phases.

**Figure 7:**
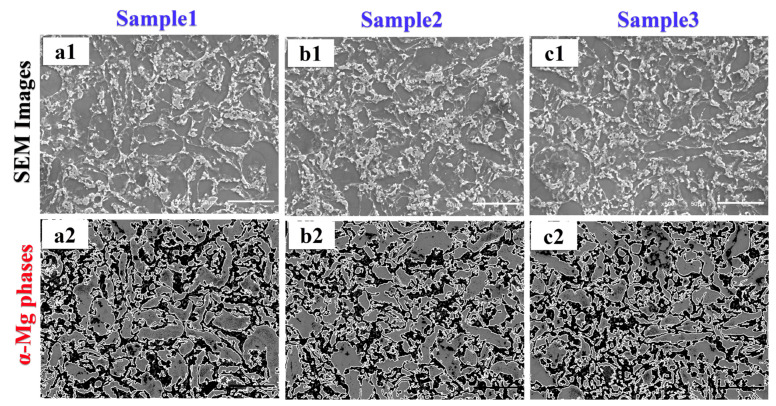
Detected grains using CV technology for samples 1, 2, and 3 (horizontal ones refer SEM images and phases; vertical ones refer Samples 1, 2 and 3; 500× magnification, scale bars with 50 µm).

The actual SEM images in Figure 7a1,b1,c1 were processed with CV technology using the proposed method. The obtained grains are indicated in Figure 7a2,b2,c2 from the actual images (Figure 7a1,b1,c1), respectively. In the SEM images, segregations are not observed in the microstructure. In the processed images (Figure 7a2,b2,c2), grey colours represent detected grains.

**Figure 8:**
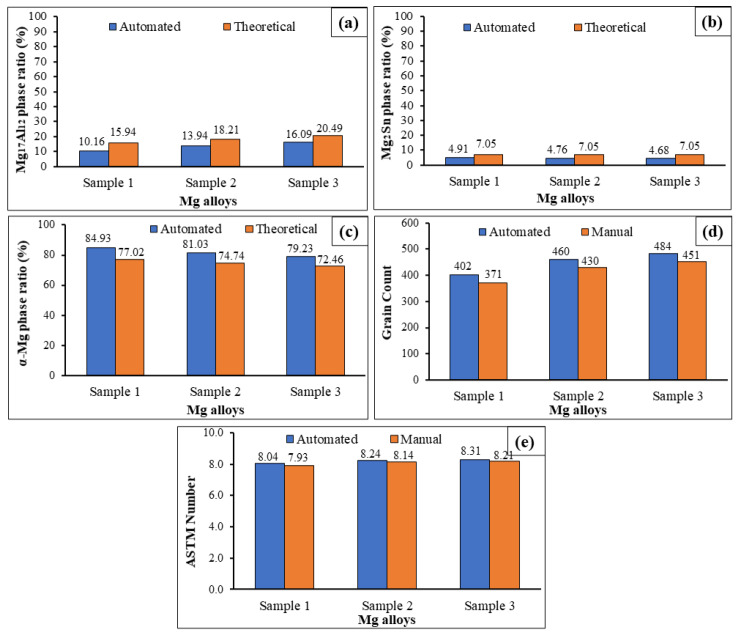
(**a**) Mg_17_Al_12_ phase ratio, (**b**) Mg2Sn phase ratio, (**c**) α-Mg phase ratio, (**d**) grain count, (**e**) ASTM grain size numbers.

According to the experiments, similar results are obtained for automated measurements and theoretical calculations. A positive correlation was found between Al content with the Mg_17_Al_12_ phases (Figure 8a). However, the correlation is negative for the α-Mg phases (grains) in Figure 8c. A Sn alloying element is equally added for samples 1–3, and changes of Mg_2_Sn phase ratios are barely observed (Figure 8b). According to the manual and automated measurements, the grain numbers increase depending on the increase of Mg_17_Al_12_ phase ratios (Figure 8d). This is also supported in other studies [35,63,67] from the literature. The grain sizes decrease with respect to the secondary phases, which act as a barrier in the grain boundaries. In the present study, it is assumed that the Mg_17_Al_12_ phases act as a barrier, and therefore the number of grains is increased per unit area. In light of the experiments, the errors of grain counting were determined as 7.71%, 6.52%, and 6.82% for Samples 1, 2, and 3, respectively. Although the sample content changes, there is little change in the error rate, and the difference between the highest and lowest error rate in the grain count is approximately 1%. It is also possible to say that the error rate decreases as the number of grains increases. Moreover, the errors of ASTM size number determining were found to be as low as 1.37%, 1.21%, and 1.20% for Samples 1, 2, and 3, respectively. The overall accuracy for determining the ASTM number is approximately 99% with automated measurements (Figure 8e). In another study [54], the Watershed Algorithm was applied to analyse grain size in the microstructure of SEM images of Ti6Al4V. The results from the Watershed Algorithm were compared with manual and MiPar measurements. The grain size values were found to be 5.18 µm, 4.72 µm, and 2.86 µm by the Watershed Algorithm, manual methods, and MiPar methods, respectively. The best result of Campbell’s study [54] for standard deviation was found to be approximately 9%.

## 4. Conclusions

This study demonstrated an automated characterization method for microstructures using SEM images with CV technology. Novel Mg alloys with different alloying element content were selected as the work materials for the experiments. The microstructure of each sample was taken using SEM imaging techniques. The intermetallic phases and grain sizes in these microstructures were counted automatically via image processing technology and corrected with manual measurements.

The obtained results were evaluated by correcting automated procedures with manual measurements. The accuracy of the automated method for determining the grain numbers was found to be over 92% compared to the manual counting procedure. The ASTM grain sizes were calculated precisely (approximately 99%) by applying CV technology according to the determined grain numbers in the SEM images. This study also offers a method to investigate the grain distributions of each alloy in the microstructure, which is a very important parameter for the metal manufacturing industry and can be used during the manufacturing process to check and qualify the material properties.

Two intermetallic phases (Mg_2_Sn and Mg_17_Al_12_) in the grain boundaries were detected in the microstructure of Mg alloys (Mg-Al-Sn). An increase in Mg_17_Al_12_ phase ratios showed an increased number of grains. Two intermetallic phases were extracted using a single SEM image input, and the outputs for each phase could be investigated separately through the image processing method.

The proposed CV method can count the grains accurately and evaluates the intermetallic phases that are present in the microstructure automatically. The method offers additional features such as measuring the areas of grain and grain boundaries to count the grain numbers, corrected with a manual method.

In short, a successful approach was proposed by calculating the ASTM grain sizes of each alloy with respect to the automated counting method. This image processing technology can be applied to many research fields due to its low input requirements. It can also be applied to other Mg alloys and different alloys such as aluminium, titanium, cobalt alloys, steels, and the like, and the results can be applied to the literature. Furthermore, CV technology offers greater capability in examining additional features without the requirement of external equipment and their laborious measurement procedures.

## Figures and Tables

**Table 1:** Summary of literature reviews of magnesium alloys and their characterizations.

Material	Alloying Elements	Synthesizing Process	Analysed Parameters	Ref
Pure Mg	Ca: 0.1–1 wt.%	Casting + Extrusion	↑ YS, ↑ UTS, ↓ GS	[17]
Mg-5Bi	Sn: 4 wt.%	Casting + Extrusion	↓ GS from 123.9 to 75.2 µm, ↑ YS by 6.63% & ↑ UTS by 8.67%	[27]
Pure Mg	Al: 8 wt.%, Sn: 2 wt.%, Zn: 1 wt.%	Casting + HPR	↓ GS, ↑ ductility	[28]
Pure Mg	Al: 3 wt.%, Zn, Mn: 1 wt.%, Ca: 0.5 wt. %	Casting	↑ S, ↑ F, ↓ GS	[29]
Mg-9Al	Mn, Ca, Sn, Y: 0.5 wt.%, Nd: 0.25 wt. %	Casting	↑ GS with Mn, Nd, Y, ↓ GS with Ca, Sn, ↓ CR with Mn, Y, Ca	[30]
Mg-Zn	Gd, Al	Casting	↓ GS, ↑ YS, ↑ UTS ↑ ductility	[31]
Pure Mg	NiTi: 0.5–3 wt.%	DMD + Extrusion	↑ D, ↓ P, ↓ GS by 76%, ↓ CTE by 10%, ↑ H by 31%, ↑ YS by 129%, ↑ UTS by 46%, ↑ EA by 35%, ↑ CYS by 104%, ↑ UCS by 26%	[32]
Pure Mg	Gd: 2–15 wt.%; Zr: 1 wt.%	Casting + Extrusion	↑ YS by 122.2%, ↓ GS from 650 to 55 µm, ↑ H, ↓CYS by 248.5%	[33]
AZ61	Mn: 0.4 wt.%; Sn: 0.8 wt.%	SLM	↓ CR, ↑ H, ↑ CS	[34]
Pure Mg	Sn: 5–13 wt.%	Hot Pressing	↑ YS, ↑ UTS	[9]
Mg-5Sn	Zn: 1–5 wt.%	Hot Pressing	↑ YS, ↑ UTS	[35]
Mg5Sn4Zn	Al: 1–4 wt.%, Mn: 0.2 wt.%	Hot Pressing	↑ YS, ↑ UTS	[36]
Mg6Sn	Zr: 0.5–2 wt.%, Mn: 0.1 wt.%	Hot Pressing	↑ YS, ↑ UTS	[26]
Mg7Sn2Zn	Mn: 0.15–0.30 wt.%	Hot Pressing	↑ YS, ↑ UTS	[25]
AM60	Ti: 1 wt.%, In: 1 wt.%, Sn: 1 wt.%	Casting + Hot rolling	↑ YS, ↑ UTS	[24]
Pure Mg	Zn: 4 wt.%, La: 1 wt.%	As-cast and As-extruded	↑ YS, ↑ UTS	[37]

D: density; P: porosity; GS: grain size; DC: damping capacity; YS: yield strength; UCS: ultimate compressive strength; PM: powder metallurgy; HPR: hard plate rolling; H: hardness; WR: wear resistance; CTE: coefficient of thermal expansion; EA: energy absorbed; UTS: ultimate tensile strength; S: strength; DMD: disintegrated metal deposition; IT: ignition temperature; BN: boron nitride; CR: corrosion rate; CYS: compressive yield strength; SLM: selective laser melting; HT: heat treatment; TID: turning-induced deformation.

**Table 2:** The chemical compositions of the Mg alloys.

Sample No.	wt.% Al, Purity: 99.9% Size: 8 µm	wt.% Sn, Purity: 99.9% Size: 10 µm	wt.% Mg, Purity: 99.8% Size: 45 µm	Sintering Temperature (°C)	Pressure (MPa)
Sample 1	7	5	Bal.	620	50
Sample 2	8	5	Bal.	620	50
Sample 3	9	5	Bal.	620	50

## Data Availability

Experimental data can be obtained by requesting them from A.E. or F.A.
